# Identification of Candidate Genes Associated with Flesh Firmness by Combining QTL Mapping and Transcriptome Profiling in *Pyrus pyrifolia*

**DOI:** 10.3390/ijms252111347

**Published:** 2024-10-22

**Authors:** Shuang Jiang, Jiaying Zhang, Xiaoqing Wang, Chunhui Shi, Jun Luo

**Affiliations:** Shanghai Key Lab of Protected Horticultural Technology, Forestry and Pomology Research Institute, Shanghai Academy of Agricultural Sciences, Jinqi Road 1000, Fengxian District, Shanghai 201403, China; jiangshuang@saas.sh.cn (S.J.); zhangjiaying@saas.sh.cn (J.Z.); wangxiaoqing@saas.sh.cn (X.W.); shichunhui@saas.sh.cn (C.S.)

**Keywords:** flesh firmness, QTL mapping, transcript, fruit development

## Abstract

Flesh firmness is an important quality of pear fruits. Breeding cultivars with suitably low flesh firmness is one of the popular pear breeding goals. At present, SNP markers related to pear flesh firmness and genes affecting flesh firmness are still uncertain. In this study, a QTL analysis was performed, and the result showed that the position of 139.857 cM in lineage group 14 (LG14) had the highest average logarithm of odds (3.41) over two years. This newly discovered locus was identified as a flesh firmness-related QTL (qFirmness-LG14). The ‘C/T’ SNP was found in corresponding Marker1512129. The ‘C’ genotype is the high-firmness genotype, which is a dominant trait. The average firmness of fruits with genotype C is 21.4% higher than genotype without the C genotype. Transcriptome profiling was obtained between ‘Zaoshengxinshui’ and ‘Qiushui’ at five time points. Three candidate genes in the interval of qFirmness-LG14 might affect firmness. A gene of xyloglucan endotransglucosylase 1 (*PpXTH1*) was upregulated in ‘Qiushui’ at all five time points. Two transcription factors (*PpHY5* and *PpERF113*) were upregulated in ‘Zaoshengxinshui’, which might be negative regulatory genes for high flesh firmness. The transcriptome results also isolated a large number of cell wall-related genes (e.g., *Pectate lyase*, *Pectin acetylesterase*, *Pectin methylesterase*, and *4-coumarate-CoA ligase*) and transcription factors (e.g., *ERF*, *WRKY*). These genes are all potential upstream and downstream genes related to flesh firmness. In conclusion, this study provides valuable insights into the QTLs and molecular mechanisms associated with fruit firmness in *Pyrus pyrifolia*.

## 1. Introduction

Fruit flesh firmness is an important quality attribute that directly impacts consumer acceptance and overall fruit quality. It is a key indicator of fruit ripeness and can influence the eating experience. Under the condition that transportation and storage of fruits are not affected, low flesh firmness better than high firmness. Pear flesh firmness is primarily determined by the composition and structure of the cell wall, which comprises cellulose, hemicellulose, and pectin [1]. These components provide structural support to the fruit and play a crucial role in maintaining firmness. As fruits ripen, the cell wall undergoes changes, such as degradation of pectin and hemicellulose, which leads to softening of the fruit flesh.

Several factors can influence fruit flesh firmness, including genetic factors, maturity stage, and environmental conditions. Genetic factors can impact fruit firmness by affecting the rate of enzymatic activity and cell wall degradation. Several studies have been conducted to understand the genetic factors influencing fruit flesh firmness in various plant species. A quantitative genetic analysis was conducted on apple fruit flesh firmness and compared physical and sensory descriptors related to this trait [2]. The genetic basis of fruit flesh firmness in apple was identified, suggesting that different cultivars have specific genes that regulate the trait of firmness [3,4]. Different cucumber cultivars also have different flesh firmness [5]. The fruit firmness of blackberry decreased noticeably at the onset of color change, and this decrease hastened from 33 days after flowering to ripening [6]. Increased watermelon fruit flesh firmness is systematically incurred with grafting on Cucurbita hybrid rootstocks [7]. The flesh firmness in tomato was influenced by drought, and a direct relationship between fruit cuticle properties, transpiration, and firmness was confirmed [8]. These studies collectively contribute to the understanding of fruit flesh firmness.

Many QTLs were related to fruit firmness. MetaQTL analysis has identified genomic loci associated with fruit quality traits, including *Md-PG1* for fruit firmness in apple [9]. In Japanese pear, QTL controlling flesh firmness have been identified in Tai4, providing valuable information for marker-assisted selection in pear breeding [10]. Genetic analysis in peach has revealed the slow-melting flesh character, with fruit firmness evolution measured over storage time [11]. In papaya, QTL conditioning desirable fruit quality traits including flesh firmness have been detected using a genotyping-by-sequencing approach [12]. Investigations in apple have identified a pectin acetylesterase gene, *MdPAE10*, contributing to prolonged fruit shelf life through QTL analysis [13]. A single QTL harboring multiple genetic variations has been linked to complicated phenotypic segregation in apple flesh firmness and crispness [14]. Promoter variations of the *ClERF1* gene have been found to influence flesh firmness in watermelon through QTL analysis [15]. Research on QTL of fruit flesh firmness in various fruit species highlights the genetic complexity and importance of understanding the mechanisms for fruit quality traits. Further studies are needed to explore additional genetic variations and regulatory pathways that contribute to fruit firmness and softening.

The genetic and molecular mechanisms of fruit flesh firmness were investigated in various fruit species. A *PECTATE LYASE5* by a *NAC* transcription factor promotes fruit softening in apple [16]. The involvement of *Md-PG1* in the fruit softening process is ethylene-dependent, as confirmed by its down-regulation with 1-methylcyclopropene treatment in apple cultivars [17]. The low expression of an *endopolygalacturonase* gene was identified in apple fruit with long-term storage potential, suggesting a potential role of this gene in fruit firmness [18]. Expression analysis was conducted in peaches with different flesh textures, and differences were identified in fruit softening and ethylene biosynthetic pathways [19]. The downregulation of specific *polygalacturonase* genes influences peach fruit texture and softening, suggesting the importance of this gene expression in determining fruit firmness [20]. Furthermore, the variation in cell wall metabolism was related to the flesh firmness of apple cultivars during fruit development, and the changes were found in cell wall thickness and intercellular space, indicating a potential link between cell wall structure and fruit firmness [16]. These studies suggest that genes related to fruit flesh firmness play a significant role in determining the flesh firmness and quality of fruits in fruit development.

In conclusion, fruit flesh firmness is a critical quality in pear. The QTLs and corresponding genes were still uncertain. In this study, our aims were to identify quantitative trait loci (QTLs) associated with flesh firmness through a genetic map and to determine candidate genes by RNA sequencing. These research findings might bring about a breakthrough in the molecular mechanism of flesh firmness and will possess significant practical implications for guiding the breeding of pear cultivars with low flesh firmness.

## 2. Results

### 2.1. QTL Mapping of Flesh Firmness

**Figure 1:**
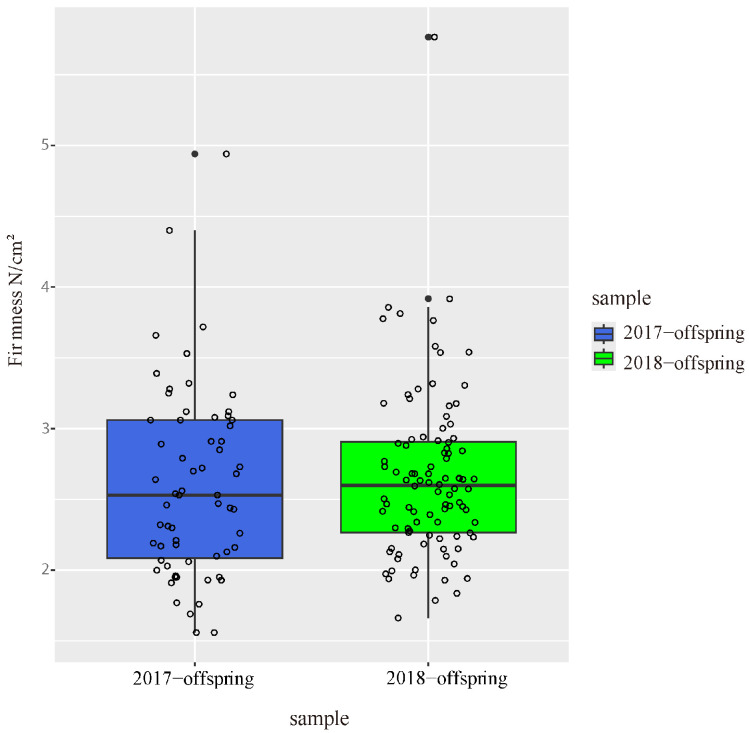
Flesh firmness of 92 offspring counted at 100 DAB in 2017 and 2018. Each dot represents a hybrid individual.

The flesh firmness of parents and offspring was evaluated at 100 DAB in two independent years. In 2017, the flesh firmness in offspring ranged from 1.56 to 4.94 N/cm^2^, and the median of flesh firmness in offspring was 2.53 (Figure 1). In 2018, the flesh firmness ranged from 1.66 to 5.77 N/cm^2^, and the median value increased slightly to 2.6. The flesh firmness of ‘Zaoshengxinshui’ was 1.56 and 2.24, respectively, in 2017 and 2018. The flesh firmness of ‘Qiushui’ was 3.28 and 3.54 respectively in 2018. There was little difference in flesh firmness between the two years.

**Figure 2:**
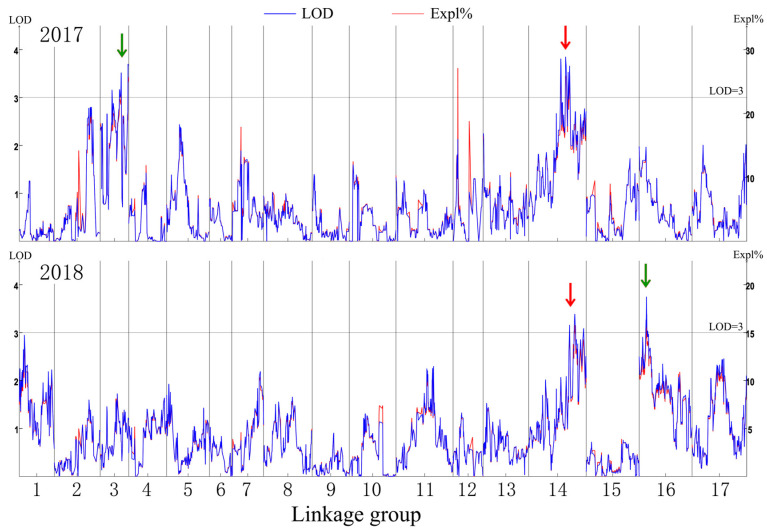
The mapping result diagram of firmness traits in 2017 and 2018. The logarithm of odds (LOD) value, marked in blue, and the explained variance (Expl %), marked in red, are shown in the left y-axis and right y-axis, respectively. The red arrows indicate a common peak, and the green arrows indicate the specific peaks in the two years.

**Figure 3:**
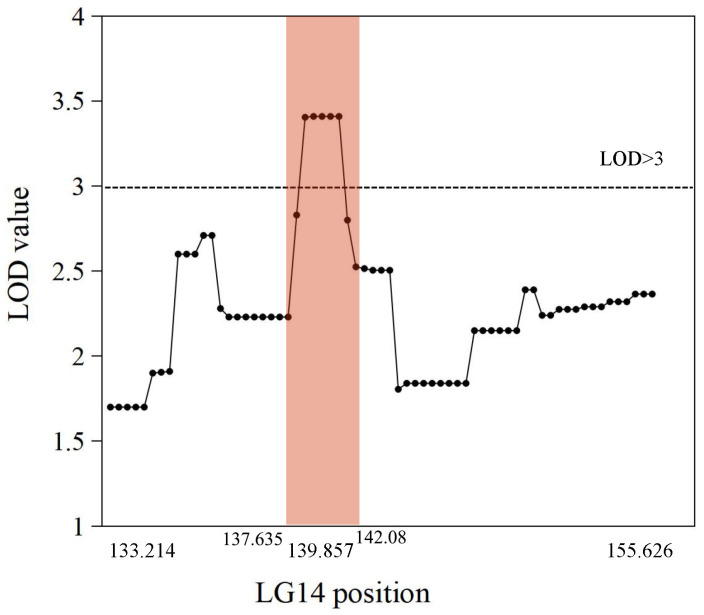
The average LOD value of markers from 2017 and 2018 data in Linkage Group 14. Each point represents a marker. The intervals of qFirmness-LG14 are labeled in peach color.

The genetic map was based on our previous study [21]. The putative QTLs for flesh firmness-related traits were identified by interval mapping at 100 days after blossom in 2017 and 2018. The logarithm of odds (LOD) value was calculated for each polymorphic site (Figure 2). In 2017, two peaks on the LOD and explained variation values were found in LG3 and LG14. The highest LOD number (3.68) was at 97.004 cM in LG3. Another obvious peak was 3.66 at 139.857 cM in LG14. In 2018, the highest number of LOD was 3.76 at 25.124 cM in LG16 and 3.16 at 139.857 cM in LG14. Although the LOD value for the two years differed in some sites, the same peak was observed in LG14. The position of 139.857 cM in LG14 had the highest average LOD (3.41) in the two years. This site was identified as a flesh firmness-related QTL (qFirmness-LG14) (Figure 3).

### 2.2. The Polymorphism of Marker1512129 in qFirmness-LG14

Five markers were found at 139.857 cM in LG14, which were Marker1512958, Marker1515613, Marker1514269, Marker1510345, and Marker1512129. In the first four markers, the genotypes of the male parent and the female parent were ‘lm × ll’. The flesh firmness was related to ‘l’, which was difficult to distinguish. The genotype of Marker1512129 in the male parent and the female parent was also ‘lm × ll’, but the flesh firmness was related to ‘m’. The SNP site information in Marker1512129 is ‘C/T’ (Figure S1). It was located at the position of 25068229 bp on the GWHBAOS00000386 fragment of the ‘Cuiguan’ pear reference genome. The ‘C’ genotype is the high-firmness genotype, and high firmness is a dominant trait. The ‘T’ genotype is the low-firmness genotype. The average firmness of fruits with genotype ‘C’ is 21.4% higher than genotype without ‘C’ (Table 1). We designed Kompetitive Allele-Specific PCR primers (Table S2) to test this site in sixteen offspring, and the test results are consistent with the re-sequencing results.

### 2.3. Gene Annotation in QTL Related Intervals

The position of qFirmness-LG14 (139.857 cM) was located on Chromosome 13 of the pear reference genome. Three positions of 137.635, 141.689, and 142.08 cM were around qFirmness-LG14. Twenty markers were isolated in these four positions. Five of them were isolated from 137.635 cM, and the remaining fifteen were from the other three positions. All markers were mapped to the pear genome. Five markers isolated from the position of 139.857 cM were located from 24,949,689 to 25,094,494 bp, which was considered as the core region. All twenty markers were located from 24,890,682 to 25,352,912 bp. Therefore, a total of 462,230 bp sequence was analyzed. After annotation, 79 genes were located in this region (Table S3). After removing uncharacterized genes and genes completely unrelated to the cell wall, 13 genes were retained, which might be key genes related to flesh firmness (Table 2).

### 2.4. Flesh Firmness in Fruit Development and RNA-Seq Analysis

**Figure 4:**
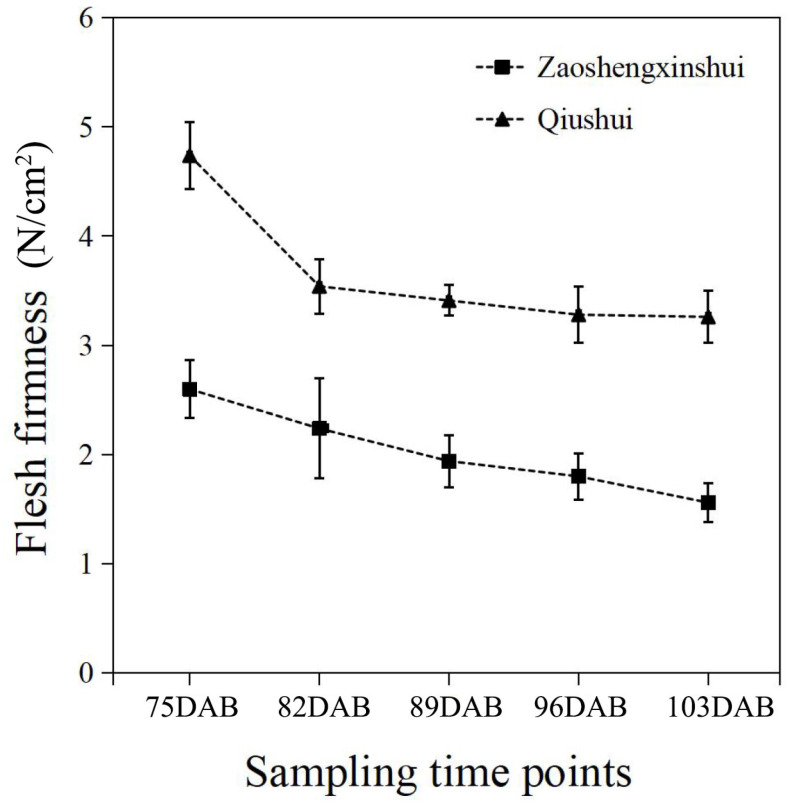
The flesh firmness of ‘Zaoshengxinshui’ and ‘Qiushui’ at five time points.

**Figure 5:**
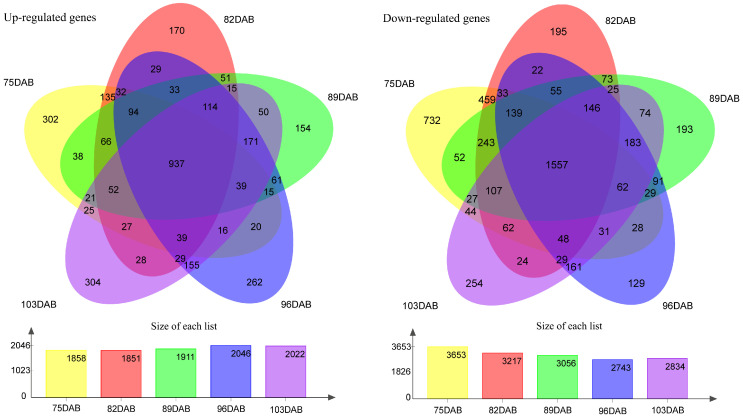
The Venn diagram of differentially expressed genes between ‘Zaoshengxinshui’ and ‘Qiushui’ at five time points. ‘Qiushui’ was used as the control cultivar; the upregulated and downregulated genes were classified in ‘Zaoshengxinshui’. The number of DEGs are shown in the column charts. Yellow, orange, green, blue, and purple represent 75, 82, 89, 96, and 103 DAB, respectively.

The flesh firmness was measured at different developmental stages of the fruit. The results showed that the firmness of flesh continuously decreases as the fruit develops (Figure 4). The firmness of the two cultivars was higher at 75 DAB than at other time points. As the fruit got closer to maturity, the downward trend of flesh firmness slowed down and approached stability in two cultivars. The flesh firmness of ‘Zaoshengxinshui’ was always lower than that of ‘Qiushui’. The flesh of ‘Zaoshengxinshui’ and ‘Qiushui’ at five time points were subjected to RNA-Seq analysis. High-throughput sequencing generated 5.77 to 7.12 GB clean reads from each library, with a Q30 percentage ranging from 91.5~92.67% (Table S4). Gene expression was compared between ‘Zaoshengxinshui’ and ‘Qiushui’ at five time points. The largest number of DEGs (5511) appeared at 75 DAB. Among them, 1858 were upregulated and 3653 were downregulated in ‘Zaoshengxinshui’ (Figure 5). The Venn diagram showed that 937 and 1557 shared genes were found in upregulated genes and downregulated genes, respectively.

### 2.5. Differentially Expressed Genes (DEGs) Related to Firmness

**Figure 6:**
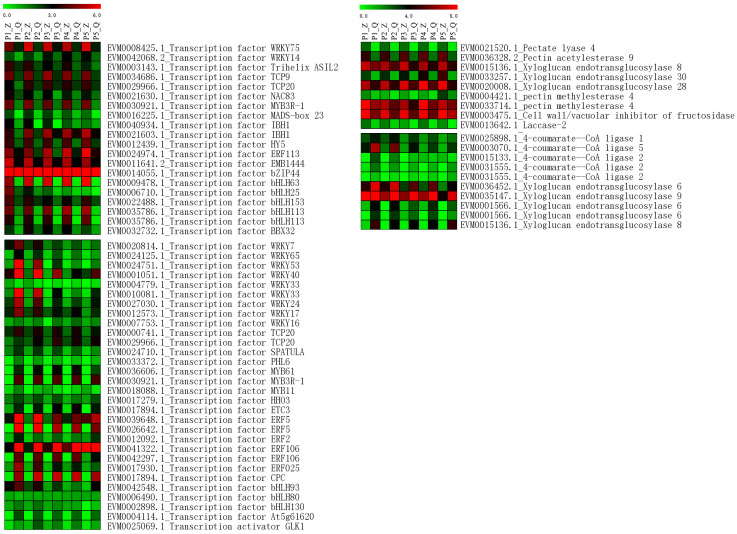
Heatmap of the expression of DEGs related to cell wall-related genes and transcription factors in fruit development. The letter ‘Z’ represents ‘Zaoshengxinshui’, and ‘Q’ represents ‘Qiushui’. P1 to P5 represent five time points (75, 82, 89, 96, and 103 DAB) for sampling. Green to red represents the gene expression level from low to high.

The 937 upregulated genes and 1557 downregulated genes were annotated by KEGG pathways (Figure S2). The ribosome pathway was found to include many genes not only in upregulated genes but also in downregulated genes. Many upregulated genes were annotated in the pathways of Biosynthesis of amino acids and carbon metabolisms, and many downregulated genes were annotated in the pathway of protein processing in the endoplasmic reticulum. According to the annotation file, we focused on searching for genes related to pectin, lignin, and cell wall. Nineteen genes were found (Figure 6). Four genes were related to pectin, which were *Pectate lyase 4*, *Pectin acetylesterase 9*, and two members of *Pectin methylesterase 4*. All of them were upregulated in ‘Zaoshengxinshui’. Five members of *4-coumarate-CoA ligase* were found to be upregulated in ‘Qiushui’. Eight members of *Xyloglucan endotransglucosylase* were found, in which three members were upregulated, and five members were downregulated in ‘Zaoshengxinshui’. *Laccase-2* and *Cell wall/vacuolar inhibitor of fructosidase* 1 were also found to be upregulated in ‘Zaoshengxinshui’. In addition to structural genes, fifty transcription factors were identified in DEGs. In upregulated genes of ‘Zaoshengxinshui’, *bHLH*, *WRKY*, and *TCP* transcription factors were found. In downregulated genes, a large number of *WRKY* and *ERF* transcription factors were isolated. The up-regulation and down-regulation expressions of these transcription factors indicate their different functions. The expression of six genes was verified through Q-PCR (Figure S3), suggesting reproducibility with the RNA-seq data.

### 2.6. Candidate Gene Expression Within Target Intervals of qFirmness-LG14

**Figure 7:**
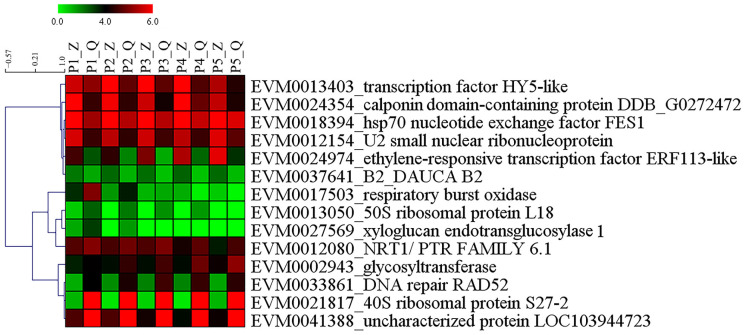
Heatmap of the expression of 14 genes related to flesh firmness around the position of qFirmness-LG14. Letter Z represents ‘Zaoshengxinshui’, and Q represents ‘Qiushui’. P1~P5 represent 75, 82, 89, 96, and 103 DAB, respectively. Green to red represents the gene expression level from low to high.

**Figure 8:**
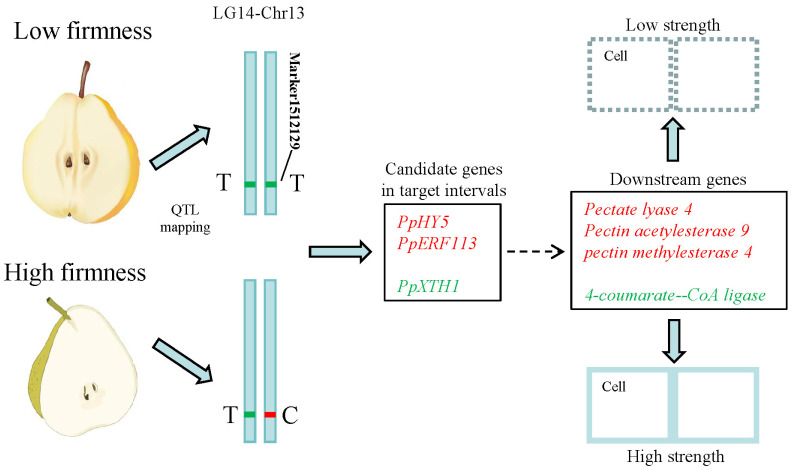
Potential regulatory pathways in pear cultivars with low and high flesh firmness. The upregulated genes in the low-firmness cultivars are labeled in red. The downregulated genes are labeled in green. ‘T’ and ‘C’ represent the genotype of Marker1512129.

A total of 79 genes were isolated within target intervals of qFirmness-LG14, and 14 DEGs were found at five time points (Figure 7). Three candidate genes were related to firmness. A gene of *Xyloglucan endotransglucosylase 1* (*PpXTH1*) was upregulated in ‘Qiushui’ at all five time points. Its expression level was relatively consistent with the firmness value. In the early stage, the expression level was very high, and as the fruit matured, the expression level decreased. *PpXTH1* was located at Chr13_25023725_25026479, which was in the core region of the position of qFirmness-LG14. Two transcription factors were found, which were transcription factor *PpHY5-like* and the *ethylene-responsive transcription factor PpERF113-like*. These two genes were upregulated in ‘Zaoshengxinshui’, which might be negative regulatory genes for flesh firmness. Based on the QTL mapping and transcriptome results, we drew a possible regulatory map (Figure 8). Three candidate genes within target intervals of qFirmness-LG14 might be the key genes for flesh firmness in *Pyrus*.

## 3. Discussion

The flesh firmness of pears was influenced by multiple aspects. When the pear is very small, the flesh is hard. During the ripening process, the cells became fragile, and the firmness was reduced. In this study, a significant drop in firmness was observed in ‘Qiushui’ at 75 DAB, and the flesh firmness of ‘Qiushui’ and ‘Zaoshengxinshui’ was decreased continuously during fruit development, which is consistent with other cultivars of pears [22], peaches [23], and loquats [24]. In the later stage of ripening, fleshy fruit softening has been mechanistically linked to cell wall disassembly.

Since flesh firmness is very important for human taste, a large number of studies have identified this trait. In *Malus*, LG01 and LG10 were observed to be related to firmness character traits [2]. Subsequent studies have shown that three QTLs for firmness were detected on LG10, LG14, and LG16 in a cross between the apple cultivars ‘Telamon’ and ‘Braeburn’. Two QTLs were detected on LG10 and LG11 in the apple cultivars ‘Orin’ and ‘Akane’ F_1_ population [25]. Multiple loci related to flesh firmness in apples indicate that there are many positions on the chromosomes associated with this trait. In *Pyrus*, a QTL was found on linkage group Tai4, detected in F1 progeny from the ‘Akiakari’ and ’Taihaku’ cross [10]. In a GWAS analysis, genotyping-by-sequencing was used to genotype 214 accessions, and a firmness QTL was found on LG16 [26]. In a high-density bin map of pears, whole-genome sequencing of pear cultivars ‘Niitaka’, ’Hongxiangsu’, and their 176 F1 progeny was performed, and more than five QTLs were identified related to flesh firmness [27]. In this study, a QTL of qFirmness-LG14 (Chr13) was found. Compared with the QTL found previously, we believe that qFirmness-LG14 is a newly discovered locus. At this site, we designed a KASP marker. The average firmness of fruits with the genotype ‘C’ is 21.4% higher than the genotype without ‘C’, which could provide theoretical guidance for high-quality pear breeding with low flesh firmness.

Three DEGs (*PpXTH1*, *PpHY5*, and *PpERF113*) were identified in target intervals of qFirmness-LG14. The genes of *XTH* and *ERF* were also found to be differentially expressed in watermelon [28], suggesting that they might be the key genes for flesh firmness. Xyloglucan endotransglucosylases/hydrolases (XTH) are enzymes that play a crucial role in plant cell wall metabolism [29]. They can catalyze the connection and rearrangement between xyloglucan molecules, thereby regulating the structure and mechanical strength of the cell wall [30]. Twenty-five *XTH* genes were found in kiwifruit and apple, and the expression levels of *Md-XTH2* and *Md-XTH10* were the highest in ripe apple fruit [31]. In tomato, the role of *XTH* during fruit growth and ripening could be related to the maintenance of the structural integrity of the cell wall, and the decrease in activity during ripening might contribute to the fruit softening [32]. The over-expression of *XTH* caused 65–84% more cell wall material to be deposited on the cross-sectional area of *Arabidopsis* hypocotyls [33]. The function of *XTH* lies in the rearrangement of polysaccharide chains during cell growth and the deposition of newly synthesized polysaccharide chains in the cell wall. In this study, *PpXTH1* was found to be upregulated in the high flesh firmness cultivar of ‘Qiushui’, suggesting that *PpXTH1* might increase the strength of cells, which improved the flesh firmness in *Pyrus*. As a key regulator in photomorphogenesis, *HY5* is involved in plant hormone signal transduction and regulation of fruit metabolism. In *Arabidopsis*, local *HY5* activity mediates hypocotyl growth and shoot-to-root communication [34]. *PyHY5* could directly bind to the promoters of *PyMYB10* and *PyWD40* and then activate their expression, thus promoting anthocyanin accumulation [35]. Our results showed that a *PpHY5* gene was upregulated in the soft flesh cultivar(‘Zaoshengxinshui’). Although *HY5* is mainly related to light, it cannot be ruled out that it might have some association with flesh firmness. *ERF* genes regulate fruit ripening by modulating processes such as pigment changes and softening in fruits [36]. During the fruit ripening process, the expression of *ERF* genes changed, which affected the physiological and biochemical processes related to fruit ripening [37], thereby influencing fruit quality and ripening times. In this study, *PpERF113* was upregulated in ‘Zaoshengxinshui’, which implied that *PpERF113* might be involved in fruit softening in *Pyrus*.

In addition to the genes from the candidate QTL interval, many cell wall-related genes were also found in the transcriptome. Three genes were related to pectin degradation. Fruit softening is the consequence of multiple cellular processes, including extensive remodeling of cell wall structure [1]. *Pectate lyase* (*PL*) can cleave the α-1,4-glycosidic bonds in pectin molecules through β-elimination, degrading the de-esterified pectin into oligosaccharides with unsaturated bonds [38]. This process disrupts the structure of pectin in the plant cell wall, making the cell wall structure of plant tissues looser, thereby leading to the softening of plant tissues [39]. In our study, the *PpPL4* gene was found to be upregulated in the soft flesh cultivar, suggesting that *PpPL4* was activated to degrade pectin in ‘Zaoshengxinshui’. *Pectin acetylesterase* (*PAE*) is able to hydrolyze the acetyl ester bonds in pectin molecules, causing pectin to undergo deacetylation [40]. The deacetylated pectin may change the porosity of the cell wall, thereby affecting cell permeability and the firmness of plant tissues, etc. This study also identified the *PpPAE9* gene, which was upregulated in the development of the soft flesh cultivar in this study. *Pectin methylesterase* (*PME*) acts on the methyl ester bonds in pectin molecules and catalyzes the demethylation reaction of pectin [41]. In the later stages of fruit ripening, further changes in *PME* activity may lead to the relaxation of the cell wall structure and the softening of the fruit. Two members of *PpPME4* were found in this study to be upregulated in the soft flesh cultivar. Our results showed that a large number of pectin degradation-related genes were found to be upregulated in the soft flesh cultivar, implying that the pectin in the soft flesh cultivar is destroyed, thereby leading to a decrease in flesh firmness. The lignin synthesis pathway was also identified in this study. Lignin is an important component of the plant cell wall. A key enzyme, 4-coumarate-CoA ligase (4CL), catalyzes the ligation reaction of 4-coumaric acid with coenzyme A to generate the corresponding coumaroyl-CoA esters. The activated coumaroyl-CoA esters are important precursor substances for lignin synthesis. Five genes of *Pp4CL* were upregulated in ‘Qiushui’ with hard flesh, suggesting that *Pp4CL* increased the lignin content in the cell wall, making the cell wall more robust. Thus, our results revealed evidence that pectin and lignin-related genes are involved in fruit softening.

Fifty differentially expressed transcription factors were found in fruit development. Some of them might be related to fruit ripening. Among them, there must be some genes related to flesh firmness. Many differentially expressed members were found among three transcription factors (*WRKY*, *bHLH*, and *ERF*) in this study. A regulatory network of *MdWRKY31*-*MdNAC7*-*MdXTH2* was found in response to ethylene signaling in apple fruit softening [42]. *PpWRKY65* could mediate peach fruit lignification [43]. *MabHLH7* positively regulated cell wall-modifying-related genes during banana fruit ripening [44]. *ERF4* affected fruit firmness through *TPL4* by reducing ethylene production in apple [45]. The *WRKY65* gene was also found to be downregulated in the cultivar with low flesh firmness. The regulatory relationship between these transcription factors and pectin/lignin still needs further study.

In conclusion, this study identified firmness-associated QTLs and analyzed the expression of related genes using transcriptome analysis. A QTL (qFirmness-LG14) for flesh firmness was mapped. A pair of KASP primers associated with qFirmness-LG14 was developed and applied to molecular marker-assisted breeding. Several differentially expressed genes related to pectin and lignin were identified. In the candidate interval of qFirmness-LG14, *PpHY5* and *PpERF113* were upregulated, and *PpXTH1* was downregulated in the soft flesh cultivar. Transcriptome analysis also showed that *PpPL4*, *PpPAE9*, *PpPME4*, and *Pp4CL* were involved in the formation of flesh firmness. This study investigated the mechanism of flesh firmness. Our new findings could provide theoretical guidance for high-quality pear production and breeding.

## 4. Material and Methods

### 4.1. Plant Materials

‘Zaoshengxinshui’ (with low average flesh firmness), ‘Qiushui’ (with high average flesh firmness), and 92 of their crossing progenies were investigated in this study. All samples were from *P. pyrifolia* and were planted in Zhuanhang Experimental Farm of the Shanghai Academy of Agricultural Sciences (Shanghai, China). In trait statistics, the fruits were collected 100 days after blossom (DAB) in 2017 and 2018, respectively. In RNA-seq analysis, the fruits of ‘Zaoshengxinshui’ and ‘Qiushui’ were sampled at 75, 82, 89, 96, and 103 DAB. At each sampling point, five fruits were collected from each sample in biological replicates. Three biological replicates were used for each experiment.

### 4.2. Evaluation of Flesh Firmness

Firstly, two pieces of 1-square-centimeter peel were removed from the pear fruit. The area after removing the peel is used to measure firmness. The average value of two pieces was the measured value for a pear fruit. Three fruits from each offspring were tested. The texture analyzer, Fruit Texture Analyser (GÜSS, Strand, South Africa), was used to conduct a puncture test. The probe speed was 1 mm/s, the probe diameter was 8 mm, and the downward pressure depth was 10 mm.

### 4.3. QTL Mapping

We constructed a genetic map based on previous research [21]. The HighMap software (1.0) was used to analyze the markers in LGs and calculate the map distances [46]. Map distances were estimated in centimorgans (cM). QTLs were detected using MapQTL software (6.0) with the phenotype data of offspring from two years [47]. A single-linkage clustering algorithm was employed to group the markers into linkage groups (LGs) by taking the independent logarithm of odds (LOD) score as a distance measure. The significance threshold for the LOD score was set at three. The genome of *Pyrus pyrifolia* ‘Cuiguan’ was used as the reference genome for pear (National Center for Biotechnology Information, https://ngdc.cncb.ac.cn/, accessed on 17 April 2024 genome version number: GWHBAOS00000000) [48].

### 4.4. RNA Extraction and RNA-Seq

The fruits of ‘Zaoshengxinshui’ and ‘Qiushui’ were processed for RNA extraction. At each time point, the flesh of five fruits was gathered for biological replicates. A total of thirty samples were examined. The CTAB method was utilized to extract total RNA from the fruit flesh. The concentration and purity of RNA was assessed using a NanoDrop 2000 spectrophotometer (Thermo Fisher Scientific, Waltham, MA, USA). Genomic DNA was digested with DNase I, and cDNA libraries were constructed following the manufacturer’s instructions using the NEBNext Ultra RNA Library Prep Kit (NEB Inc., Boston, MA, USA). The quality of the library was evalufated using an Agilent Biological Analyzer 2100 system (Agilent, Palo Alto, CA, USA). Paired-end sequencing was carried out using the Illumina HiSeq 4000 system (Illumina, San Diego, CA, USA). Preprocessing filters and trims of the raw data were applied to eliminate reads containing adapters, reads with more than 5% unknown nucleotides, and low-quality reads with more than 20% of bases having a quality value ≤10. Only clean reads were employed in the subsequent analyses.

### 4.5. Identification of Significantly Differentially Expressed Genes (DEGs)

The HISAT software (2.0) was employed to align the filtered reads to the reference pear genome [49]. To ensure comparability of gene expression levels between different genes and different samples, fragments per kb per million reads (FPKM) was used to standardize the expression level. DESeq2 was utilized to conduct a differential gene expression analysis. The threshold for determining the significance of gene expression differences was set to |log2FoldChange| > 1 and a significance *p* value < 0.05. ‘Qiushui’ was used as the control cultivar; the upregulated and downregulated genes were classified in ‘Zaoshengxinshui’. For KEGG pathway analysis, all differentially expressed genes (DEGs) were first mapped to KEGG terms in the database (https://www.genome.jp/kegg/pathway.html, accessed on 12 June 2024), and the gene numbers for each term were calculated to identify significantly enriched KEGG terms in the input list of DEGs. A heatmap of gene expression was obtained using MEV 4.0.

### 4.6. Kompetitive Allele Specific PCR (KASP) Primer Design and Detection

Polymorphic sequences of Marker1512129 (C/T SNP) were submitted to LGC Biosearch Technologies (LGC, London, UK) to order the KASP Assay Mix, which typically consists of three KASP primers to a SNP site. The PCR mixture, with a total volume of 10 μL, contained 5 μL of 2xKASP-TF Master Mix (LGC-KBS-1050-121, LGC, London, UK), 5 μL DNA, and 0.14 μL of KASP Assay Mix. The three-step program started at 94 °C for 15 min, followed by 9 cycles of 94 °C for 20 s, and 61 °C for 60, followed by 25 cycles of 94 °C for 20 s, and 55 °C for 60s. The reactions were carried out on a LightCycler 480 instrument (Roche, Basel, BS, Switzerland). The PCR products were tested using ‘Endpoint Genotyping Analysis’.

### 4.7. Expression Analysis of Quantitative Real-Time PCR

For the two pear cultivars (‘Zaoshengxinshui’ and ‘Qiushui’), RNA was extracted at five time points. The PrimerScript RT Reagent Kit with gDNA Eraser (RR047, Takara, Osaka, Japan) was used to synthesize first-strand cDNA. The real-time PCR mixture, with a total volume of 15 μL, contained 7.5 μL of TB Green Premix Ex Taq (RR420A, Takara, Osaka, Japan), 1 μL of each primer (10 μM), 1 μL of cDNA, and 4.5 μL of double-distilled water. The reactions were carried out on a LightCycler 480 instrument (Roche, Basel, Switzerland). The two-step Q-PCR program started at 95 °C for 30 s, followed by 40 cycles of 95 °C for 5 s and 60 °C for 30 s. The expression was calculated as 2^−ΔΔCt^ and normalized to the expressions of UBI (XM_009368893.2) and the actin gene (JN684184). All the primers used are listed in Table S1.

## Figures and Tables

**Table 1:** Firmness statistical results corresponding to the genotypes of 94 offspring.

Marker	Genotype	Phenotype Flesh Firmness (N/cm^2^)	Number of Offspring
Marker 1512129	CT	2.89	43
	TT	2.38	41

**Table 2:** The annotation of genes related to QTL of qFirmness-LG14.

‘Cuiguan’ Genome	NR Description	Position
EVM0024354	calponin homology domain-containing protein DDB_G0272472	Chr13_24912662_24915561
EVM0013403	transcription factor HY5-like	Chr13_24920364_24922189
EVM0013050	50S ribosomal protein L18	Chr13_24949033_24950857
EVM0002943	glycosyltransferase	Chr13_24961143_24963335
EVM0033861	DNA repair RAD52	Chr13_24967729_24970208
EVM0018394	hsp70 nucleotide exchange factor FES1	Chr13_24971327_24973746
EVM0012154	U2 small nuclear ribonucleoprotein	Chr13_24981861_24985672
EVM0027569	xyloglucan endotransglucosylase 1	Chr13_25023725_25026479
EVM0017503	respiratory burst oxidase	Chr13_25059679_25063772
EVM0021817	40S ribosomal protein S27-2	Chr13_25208357_25209651
EVM0012080	NRT1/PTR FAMILY 6.1	Chr13_25258968_25261202
EVM0024974	ethylene-responsive transcription factor ERF113-like	Chr13_25312766_25315579
EVM0037641	B2_DAUCA B2	Chr13_25333710_25334935

## Data Availability

The RNA-seq data was deposited in the National Genomics Data Center, China National Center for Bioinformation/Beijing Institute of Genomics (BIG), Chinese Academy of Sciences. The run accession No. of RNA-seq data was CRR592869-CRR592874, CRR592877-CRR592882, CRR592885-CRR592890, CRR592893-CRR592898, CRR592901-CRR592906. The rest of the data supporting our findings are contained within the manuscript and Supplementary Files.

## Supplementary Materials

The following supporting information can be downloaded at: https://www.mdpi.com/article/10.3390/ijms252111347/s1.
