# Supplementary figures and images for: Identification of Candidate Genes Associated with Flesh Firmness by Combining QTL Mapping and Transcriptome Profiling in Pyrus pyrifolia
Source: Int J Mol Sci. 2024 Oct 22;25(21):11347. doi: 10.3390/ijms252111347 (PMC11545808; doi:10.3390/ijms252111347)

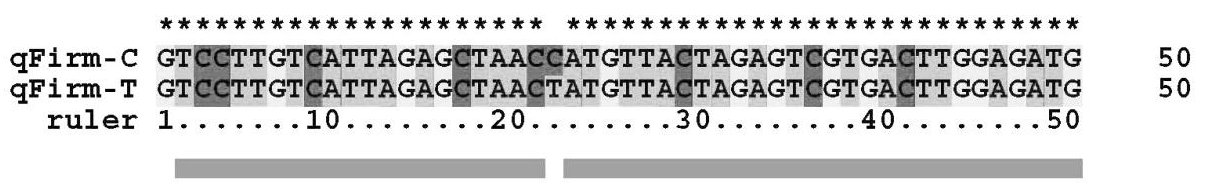

Supplement: Supplementary file 1 [file ijms-25-11347-s001.zip › Figure S1 Gene sequence comparison results at the site of Marker1512129.png]

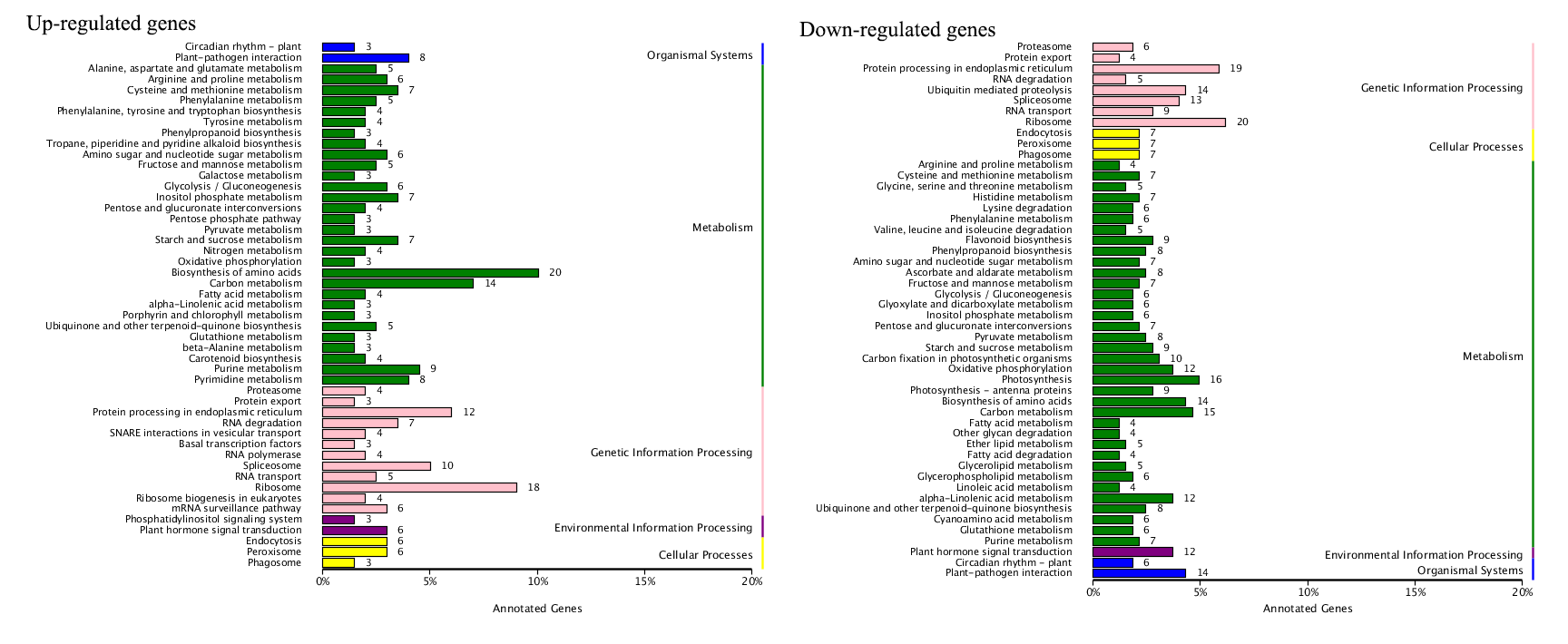

Supplement: Supplementary file 1 [file ijms-25-11347-s001.zip › Figure S2 KEGG enrichment map of differentially expressed genes.png]

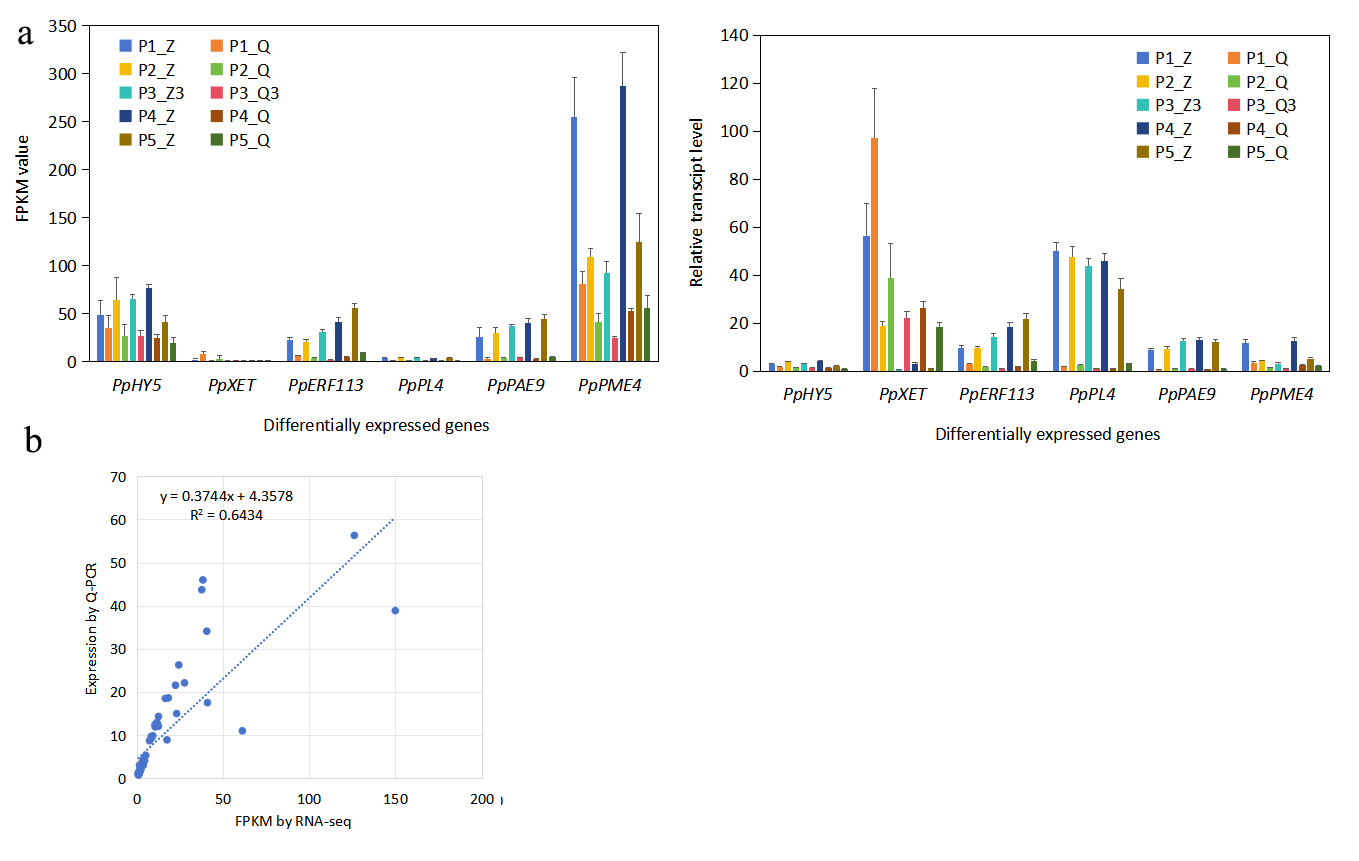

Supplement: Supplementary file 1 [file ijms-25-11347-s001.zip › Figure S3 The expression of six genes in ‘Zaoshengxinshui’ and ‘Qiushui’ at five time points. (a) Q-PCR validation. (b) Coefficient analysis between the gene expression ratios obtained from RNA-seq and Q-PCR data.png]
